# Assessment of the Antiangiogenic and Anti-Inflammatory Properties of a Maslinic Acid Derivative and its Potentiation using Zinc Chloride

**DOI:** 10.3390/ijms20112828

**Published:** 2019-06-10

**Authors:** Ioana Zinuca Pavel, Rene Csuk, Corina Danciu, Stefana Avram, Flavia Baderca, Andreea Cioca, Elena-Alina Moacă, Ciprian-Valentin Mihali, Iulia Pinzaru, Danina Mirela Muntean, Cristina Adriana Dehelean

**Affiliations:** 1 Department of Pharmacognosy, Faculty of Pharmacy, “Victor Babeş” University of Medicine and Pharmacy, 2, Eftimie Murgu Sq., Timişoara 300041, Romania; ioanaz.pavel@umft.ro (I.Z.P.); stefana.avram@umft.ro (S.A.); 2 Department of Organic Chemistry, Martin-Luther University Halle-Wittenberg, Kurt-Mothes-Str. 2, D-06120 Halle (Saale), Germany; rene.csuk@chemie.uni-halle.de; 3 Department of Microscopic Morphology, Faculty of Medicine, "Victor Babeş" University of Medicine and Pharmacy, 2, Eftimie Murgu Sq., Timişoara 300041, Romania; baderca.flavia@umft.ro; 4 Department of Pathology, CFR Clinical Hospital, 13−15, Tudor Vladimirescu, Timişoara 300173, Romania; cioca.andreea19@gmail.com; 5 Department of Toxicology, Faculty of Pharmacy, "Victor Babeş" University of Medicine and Pharmacy, 2, Eftimie Murgu Sq., Timişoara 300041, Romania; alina.moaca@umft.ro (E.-A.M.); iuliapinzaru@umft.ro (I.P.); cadehelean@umft.ro (C.A.D.); 6 “George Emil Palade” Electron Microscopy Center, Institute of Life Sciences, Faculty of Medicine, “Vasile Goldiș” Western University of Arad, 86, Liviu Rebreanu St., Arad 310414, Romania; mihaliciprian@uvvg.ro; 7 Department of Functional Sciences - Pathophysiology, Faculty of Medicine, "Victor Babeş" University of Medicine and Pharmacy, 2, Eftimie Murgu Sq., Timişoara 300041, Romania; daninamuntean@umft.ro; 8 Center for Translational Research and Systems Medicine, "Victor Babeş" University of Medicine and Pharmacy, 2, Eftimie Murgu Sq.,Timişoara 300041, Romania

**Keywords:** EM2, benzylamide derivative, maslinic acid, zinc chloride, inflammation, angiogenesis

## Abstract

Maslinic acid is a pentacyclic triterpene with a plethora of biological activities, including anti-inflammatory, antioxidant, antimicrobial, cardioprotective, and antitumor effects. New derivatives with improved properties and broad-spectrum activity can be obtained following structural changes of the compound. The present study was aimed to characterize a benzylamide derivative of maslinic acid—benzyl (2α, 3β) 2,3-diacetoxy-olean^−^12-en-28-amide (EM2)—with respect to the anti-angiogenic and anti-inflammatory effects in two in vivo experimental models. Consequently, the compound showed good tolerability and lack of irritation in the chorioallantoic membrane assay with no impairment of the normal angiogenic process during the tested stages of development. In the acute ear inflammation murine model, application of EM2 induced a mild anti-inflammatory effect that was potentiated by the association with zinc chloride (ZnCl_2_). A decrease in dermal thickness of mice ears was observed when EM2 and ZnCl_2_ were applied separately or in combination. Moreover, hyalinization of the dermis appeared only when EM2 was associated with ZnCl_2_, strongly suggesting the role of their combination in wound healing.

## 1. Introduction

Extensive experimental research was performed in recent years in the field of drug discovery in the effort to find natural therapeutic approaches for the management of severe conditions such as cancer.

Investigating the enormous potential of the Plant Kingdom represents an attractive alternative for drug development [1]. Indeed, medicinal plants as such or in the form of total/ standardized extracts or pure isolated phytochemicals, still represent today one of the main sources for the identification and development of novel active molecules [2]. Importantly, phytochemicals within an extract can synergistically act and simultaneously target different mechanisms of action or signal transduction pathways, thus improving their therapeutic efficacy [3,4]. Nevertheless, in order to be approved by regulatory authorities, as in the case of synthetic molecules, an ample chemical characterization together with rigorous pharmacology and toxicology studies are required [5].

Pentacyclic triterpenes represent one of the highly investigated group of phytochemicals. This class possesses various therapeutic effects, being used in numerous diseases including cancer [6,7,8]. Maslinic acid is mainly obtained from the fruits of *Olea europaea* L. and is a representative pentacyclic triterpene with a wide range of biological activities [9,10]. In order to determine the safety of maslinic acid, Sanchez-Gonzalez et al. evaluated the effect of the compound after acute and repeated oral administration in mice [11]. Haematological and biochemical assays revealed neither differences nor side effects as compared to the untreated mice, thus indicating that the compound can be considered a safe molecule [11].

New derivatives with improved physico-chemical properties and broad-spectrum activity can be obtained following structural changes of a compound [12] and an intensive research effort is dedicated to find the perfect structure-activity relationship [13].

Maslinic acid, an attractive and safe pentacyclic triterpene with multiple biological activities [14] has been reported, among others, to modulate angiogenesis [15,16]; however, the number of experiments is limited and the available results were obtained from in vitro assays. To the best of our knowledge, no evaluation of benzyl (2α, 3β) 2,3-diacetoxy-olean^−^12-en-28-amide (EM2) with respect to the angiogenic process has been previously described.

The chorioallantoic membrane (CAM) assay is successfully used in various biologic experiments and is especially useful in tumor and angiogenesis studies [17,18] as an alternative prescreening method to the tumor mouse models. This assay has several advantages, as it is low cost, less time consuming, and can be done in simple practical setting [19].

In a recent review, Danciu et al. [20] pointed out data concerning the liaison between angiogenesis and inflammation, one of the common mechanisms being related to oxidative stress and the release of cytokines activating a series of transcription factors that can modulate inflammation and act as promoters of angiogenesis.

Rudolf Virchow was among the first to indicate a connection between inflammation and cancer [21]. Since then, concepts have evolved to the current understanding that inflammation has a crucial role in tumor initiation and promotion [22,23]. An increased expression of oncogens and pro-inflammatory transcription factors and an elevated level of pro-inflammatory mediators contribute to tumor proliferation, invasion, metastasis, and also to radioresistance and chemoresistance [23].

Numerous plants derived compounds were found to reduce chronic inflammation, including the class of pentacyclic triterpenes [21,24,25,26].

Zinc is an essential microelement that was studied in topical preparations to diminish inflammatory skin conditions, acne, and hair loss [27].

The experimental model of 12-o-tetradecanoylphorbol^−1^3-acetate (TPA)-induced ear inflammation is a useful tool to assess the anti-inflammatory potential of newly obtained compounds; it is considered easy to perform and it rapidly provides data regarding the beneficial effects or toxic activity of topical applied formulations [28].

To the best of our knowledge, no literature data are available that describe the effect of EM2 alone or in association with zinc chloride (ZnCl_2_) using the TPA-induced local acute inflammation model.

The present study was aimed to: (i) characterize a benzylamide derivative of maslinic acid (EM2); (ii), which would evaluate the effects of EM2 on the angiogenesis process using the CAM assay; and iii) to evaluate the effects of different EM2 formulations for topical application (given alone EM2 1% hydrogel) or in association with zinc chloride (1% and 5% hydrogels) in an experimental model of acute local murine inflammation induced by the topical application of TPA on the ear.

## 2. Results

### 2.1. Transmission Electron Microscopy (TEM) Analysis of EM2

**Figure 1:**
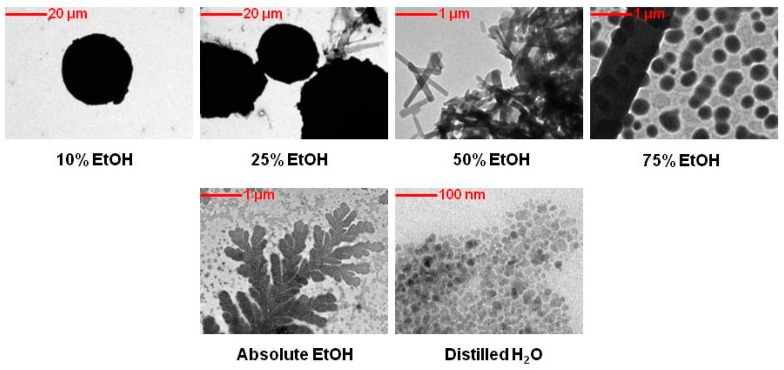
Transmission electron microscopy (TEM) images of benzyl (2α, 3β) 2,3-diacetoxy-olean^−1^2-en-28-amide (EM2).

Figure 1 displays TEM images of EM2 in different concentrations of hydroalcoholic solutions. It can be noticed that with increasing the ethanol concentration in aqueous solutions the morphology of the microparticles change. As such, at low aqueous alcoholic concentration solutions (10% and 25%), the morphology of EM2 microparticles was sphere like with an average size of 12 µm. At high ethanol concentrations (50% and 75%), the morphology of the microparticles varied from circular shapes to irregular needle-shaped structures. Furthermore, when the microparticles were dissolved in absolute ethanol, crystallization occurred, with acicular crystals organized in a ‘leaf’ shape being observed.

In order to verify the behavior and morphology of these microparticles, they were dissolved in distilled water. It can be noted that in distilled water the microparticles of EM2 are clustered together, having irregular shapes with faceted microparticles forming micro aggregates.

### 2.2. Scanning Electron Microscope (SEM) Analysis of EM2

**Figure 2:**
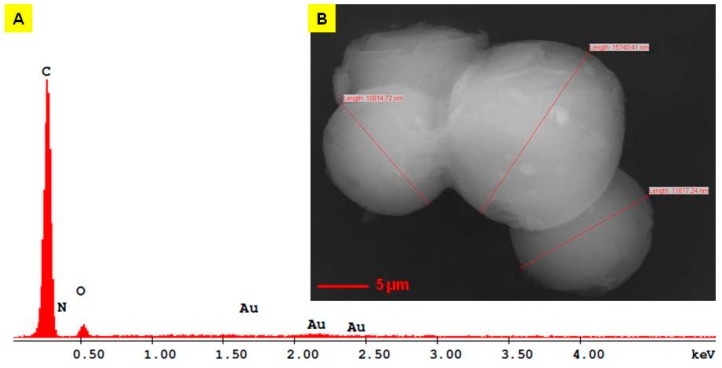
Energy dispersive X-ray spectra (**A**) and scanning electron microscope (SEM) image of EM2 (**B**) [29].

A SEM image of EM2 is present in Figure 2B. The SEM data is in accordance with the TEM measurements with respect to particle size; the diameters measured using SEM are between 10.81 and 15.74 μm. The morphology of the EM2 surface is characterized by the agglomeration of the microparticles with an irregular and sphere like shape. In high vacuum mode, it is observed that the facets of microparticles are in contact with one another and between them are spherical microparticles lower in size.

**Figure 3:**
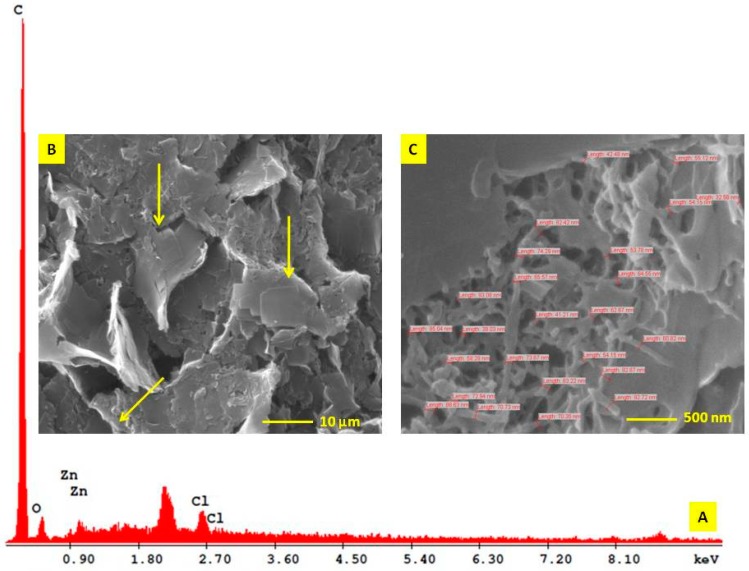
Energy dispersive X-ray spectra (**A**) and SEM image of EM2+ZnCl_2_; (**B**) – rough surface overview aspect with square shape ultrastructures – yellow arrows; (**C**) – trabecular aspect of square shape ultrastructures in a fracture area.

On the other hand, EM2 + ZnCl2 5% examined using SEM showed a unitary rough surface morphology structure composed predominantly of square shape ultrastructures (Figure 3B, yellow arrows). Under 80K magnification, one can notice in a fracture area inside of these square surfaces a trabecular grid formed by filaments of 66 nm in size (Figure 3C).

The EM2 + ZnCl2 5% compound was obtained in a mass ratio of 1:5 in a hydro-alcoholic solution. The resulted compound had a hard-unitary structure organized in macroscopic domains, which makes it not suitable for a proper TEM examination (with respect to the largest sample size expressed in µm).

### 2.3. Energy Dispersive X-ray Analysis (EDAX) of EM2

For the determination of the elemental composition of EM2 and of the EM2 + ZnCl2 mixture, the energy dispersive X-ray analysis (EDAX) was performed (Figure 2A and Figure 3A). The only elements present in the EM2 sample were C, O, and N. The presence of gold in the EDAX spectrum was derived from the preparation of the sample for examination. The microparticles were covered with gold just to be able to join the analysis grid. An AutoAgar sputter coater with a sputtering deposition rate of 3 nm gold thickness/10 sec cycle for three times was used (Table 1).

Regarding the EM2 + ZnCl2 mixture, the EDAX analysis revealed that the mixture has a simple composition represented by C, O, Zn, and Cl, expressed in Wt% and At% (Table 2).

### 2.4. Thermogravimetry-Differential Scanning Calorimetry (TG-DSC) Analysis of EM2

**Figure 4:**
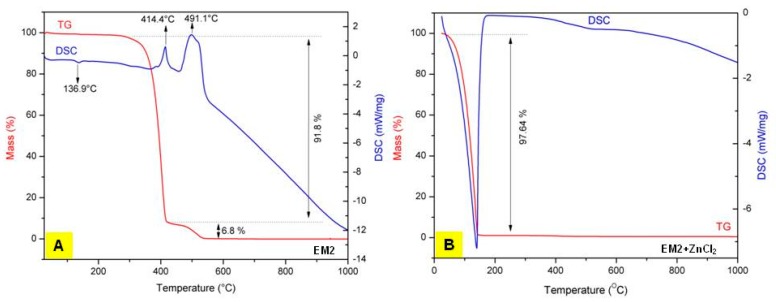
Thermogravimetry-differential scanning calorimetry (TG-DSC) curves of **A**: EM2 [29] and of the **B**: EM2 + ZnCl2 mixture.

Figure 4 displays the TG-DSC curves of EM2 and of the EM2 + ZnCl2 mixture, respectively. Regarding the TG-DSC curves of EM2 (Figure 4A), on the differential scanning calorimeter (DSC) curve one can observe a weak endothermic effect at 136.9 °C. This endothermic effect, without mass loss, corresponds to the melting of EM2. On the other hand, on the TG curve can be noticed an exothermic effect at 414.4 °C accompanied by an important mass loss of 91.8%. This exothermic effect can be imputable to the oxidative degradation of EM2. Likewise, another exothermic effect can be observed on the TG curve, which is stronger than the first one, between 475–550 °C, with a mass loss of 6.8%. This exothermic effect is assigned to the carbon burning, which is present in the EM2 compound.

In order to investigate the thermal behavior of the hydro-alcoholic solution of EM2 + ZnCl_2,_ the mixture was partially dehydrated in an oven at 70 °C for 1 h. The only effect observed for the hydro-alcoholic mixture on DSC curves was an endothermic one after 100 °C, associated with a significant mass loss registered on TG curve, an effect which corresponds to the evaporation of the solvent used to dissolve EM2 and ZnCl_2_ (Figure 4B).

### 2.5. EM2 Effects on Normal CAM

No signs of hyperemia, bleeding, or coagulation phenomena were noted throughout the experiment. EM2 was well tolerated and good viability of the specimens were registered, having similar survival rates with the control group until embryonic day of development (EDD) 13 when the specimens were sacrificed.

Vessel development and architecture was not impaired by the application of EM2. Compared to the control specimens, there was no influence upon the number of small new forming vessels. EM2 applied during a highly angiogenic interval indicated that this compound supports the normal functionality of the vascular pattern of the membrane.

**Figure 5:**
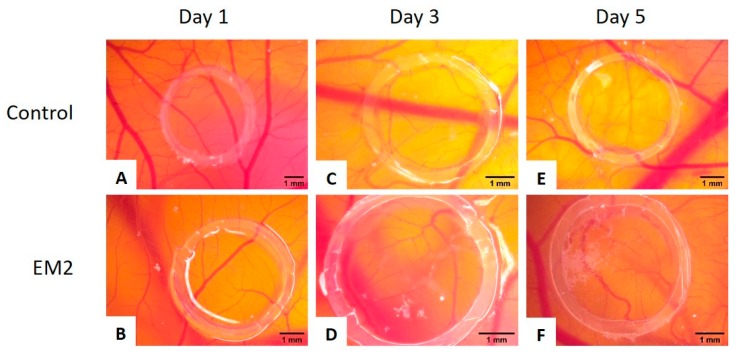
Normal chick chorioallantoic membrane-angiogenesis evaluation of EM2; suggestive stereomicroscopic images were registered on day 1 (**A**,**B**), 3 (**C**,**D**) and 5 (**E**,**F**) of the experiment: control containing only 1% dimethyl sulfoxide (DMSO) (**A**,**C**,**E**) and 100 µM EM2 (**B**,**D**,**F**) [29].

The early sprouting angiogenesis, up to day 3 (EDD 11), was not influenced (Figure 5D). From day 3 to day 5, an increased number of tertiary blood vessels were present around the administration spot, forming a honeycomb like pattern, easily noticeable on the final day of the experiment (Figure 5F). The effect was similar to the control specimen (Figure 5E), revealing a normal ongoing vascular branching after incubation with EM2 (Figure 5F). After five doses of EM2, a limited number of capillary dilatations were observed outside the application ring converging toward the ring with tortuous aspect. Moreover, when comparing to the control samples, where some hemorrhagic spots could be signaled, the EM2 treated specimens were hemorrhage free (Figure 5F), indicating a potential anti-inflammatory, vessel protective activity.

**Figure 6:**
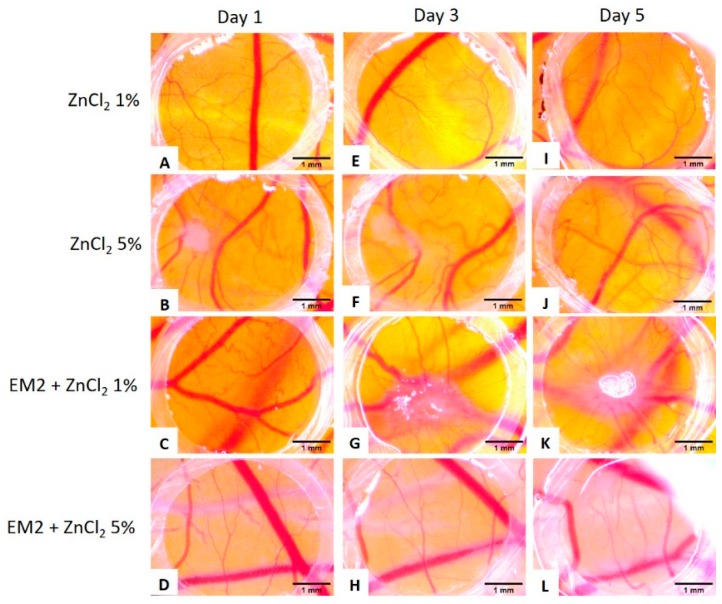
Normal chick chorioallantoic membrane-angiogenesis evaluation of ZnCl_2_ and of EM2 + ZnCl_2_; suggestive stereomicroscopic images were registered on day 1 (**A**–**D**), 3 (**E**–**H**) and 5 (**I**–**L**) of the experiment. EM2 100 µM and ZnCl2 1% and 5% (*m*/*m*) were tested.

The effect of ZnCl_2_ on the developing CAM was also evaluated, and both tested concentrations of ZnCl_2_ were tolerated (Figure 6A,B,E,F,I,J). Slight modifications of the vessel architecture was observed, with fewer new formed capillaries, a lower degree of branching pattern, and few micro-hemorrhages that were in remission by day 5 (Figure 6I,J).

The combination between EM2 and ZnCl_2_ were also tested and no sign of toxicity was observed. For the combination with ZnCl_2_ 1% on the spot of the inoculation, there was a moderate vascular reaction, but the number of small capillaries and branching were low, inferior to those in untreated areas (Figure 6C,G,K). The combination with ZnCl_2_ 5% was even better tolerated, showing no major sign of impairment on the vascular architecture, with a tendency for fewer branches and narrower capillaries compared to untreated CAM areas (Figure 6D,H,L).

### 2.6. Evaluation of EM2 and ZnCl_2_ Formulations Topic Application

Regarding the experimental mice model of inflammation, ears evaluation with the electronic calliper denoted that the groups treated with EM2 1% hydrogel or with EM2 and ZnCl_2_ hydrogels (groups 6, 9, and 10) presented a reduction in ear edema, compared to the group treated with Blank hydrogel (group 5). After application of the TPA solution, a visible increase in mice ear edema could be spotted. Treatment with indomethacin elicited a significant decrease of ear inflammation and redness. Blank hydrogel application did not reduce ear edema or redness; the results obtained in this case are comparable with the ones obtained following the TPA solution application.

The groups treated with ZnCl_2_ hydrogel (groups 7 and 8) showed a mild reduction of ear edema and inflammation and a moderate anti-inflammatory effect when a higher concentration was used.

Mice ears exposed to TPA solution and then treated with EM2 + ZnCl_2_ hydrogels showed similar macroscopic aspects as the group treated with EM2 1% hydrogel alone.

Histopathological evaluation was performed in order to see changes within the tissue induced using the pro-inflammatory agent and screened treatment.

### 2.7. Assessment of the Histopathologic Analysis of Mice Ears

The mice were anesthetized and sacrificed at the end of the study. Ears were collected in order to perform the histopathological analysis. Hematoxilin-eosine (H-E) was used to stain the tissue samples.

**Figure 7:**
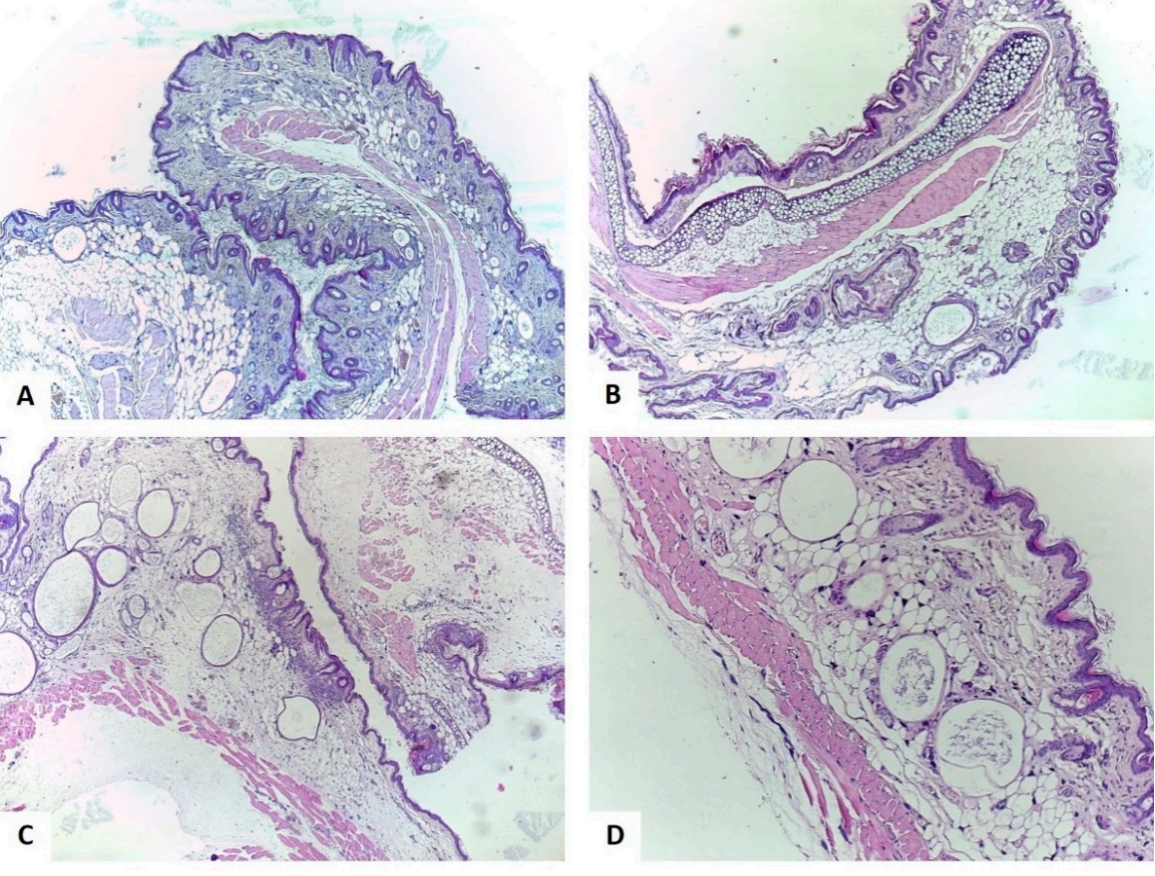
Histological aspects of the skin hematoxilin-eosine (H-E) stain, **A**: Control group – with no intervention, ob. 4×; **B**: Acetone group, ob. 4×; **C**: TPA group, ob. 4×; **D**: TPA + indomethacin group, ob. 10×. [29].

The skin from the control group was formed of 3–4 layers of keratinocytes and 4–5 thin filaments of keratin (Figure 7A). The mast cells were the only inflammatory cells present, but it is considered a common finding in the skin specimens of SKH1 mice. The mast cells were filled with basophilic granules, big and diffusely disposed in the dermis and in the perifollicular and perianexial areas (Figure 7A).

Following acetone application, marked dermal interstitial edema without signs of inflammation could be detected (Figure 7B).

As presented in Figure 7C, TPA had a significant pro-inflammatory action on mice skin. The epidermis was composed of nine layers of keratinocytes, it was consistently enlarged, and a thick layer of keratin filaments were disposed over it. Under the epidermis, an abundant band like inflammatory infiltrate composed of neutrophils was observed. In addition, intraepidermal abscesses were formed by the neutrophils that migrated in the epidermal thickness (exocytosis). In the dermis, almost no mast cells could be noted; if there were any, they were probably distributed in the reticular dermis. A handful of blood vessels showed mild congestion; some also displayed leukocytes margination. At the dermal level, no hyalinization was noted.

Application of indomethacin (Figure 7D) lead to a reduction of the inflammatory process compared to the TPA group, the inflammatory cells being formed by a moderate number of neutrophils. No intraepithelial abscesses were present. In the reticular dermis only a small number of mast cells could be observed. Compared to the TPA group, the interstitial edema was reduced and dermal hyalinization appeared.

**Figure 8:**
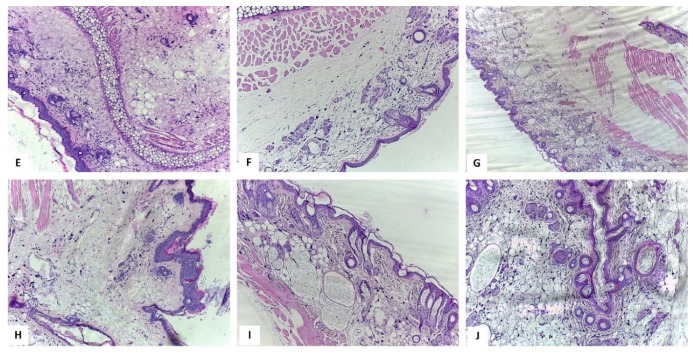
Histological aspects of the skin H-E stain, **E**: Blank hydrogel group, ob. 10× – the sample displayed inflammatory cells distributed in the entire dermis and massive interstitial edema; **F**: ZnCl_2_ 1%, ob. 10× – Reduced number of neutrophils and moderate interstitial edema; **G**: ZnCl_2_ 5%, ob. 4× – Reduced number of neutrophils and dermis hyalinization; discreet edema and mild congestion of the blood vessels; **H**: EM2 1% group, ob. 10× – in the entire dermis mild inflammation and moderate interstitial edema were observed; **I**: EM2 + ZnCl_2_ 1%, ob. 10× – Hyalinization of the dermis and epidermal alterations were similar to the group treated with ZnCl_2_ 1%. **J**: EM2 + ZnCl_2_ 5%, ob. 10× - Hyalinization of the dermis and epidermal alterations were similar to the group treated with ZnCl_2_ 5%. [29].

Application of the Blank hydrogel induced the enlargement of epidermis because of acanthosis (nine layers of keratinocytes) and orthokeratosis with 2–3 thick keratin filaments that covered the epidermis (Figure 8E). The dermis was also enlarged due to massive interstitial edema. Nevertheless, in this group the inflammation was reduced and, in the dermis, a medium number of neutrophils were diffusely distributed. The mast cells were small, being fewer than in the Control group and were disposed in the deep dermis.

Figure 8F presents the skin specimen of mice treated with ZnCl_2_ 1%—the epidermis presented acanthosis and hypergranulosis, being formed of 6–7 layers of keratinocytes covered by 5–6 keratin filaments (hyperorthokeratosis). Neutrophils were disposed in the dermis, indicating a mild inflammatory infiltrate. Furthermore, mast cells were also observed but were smaller than in the Control group and were disposed in all levels of the dermis. Moderate interstitial edema was also noted; the superficial dermis was hyalinized.

In the case of mice treated with the higher concentration of ZnCl_2_, namely 5% (Figure 8G), the epidermis had acanthosis with 6–7 layers of keratinocytes covered by 3–4 filaments of keratin. The mast cells were similar in size and number to the control group, but in the case of ZnCl_2_ 5% treated mice the distribution of the cells was different, the cells being disposed in the reticular and superficial dermis; scattered neutrophils were also observed in this group. Leukocytes margination was noted in a few blood vessels; discreet interstitial edema and reduced hyperemia were also observed. The reticular and superficial dermis were hyalinized.

The skin specimen of the group with topical application of EM2 1% is presented in Figure 8H. In this case, the epidermis was focally enlarged compared to the untreated group. A mild inflammatory infiltrate was observed that was formed of neutrophils diffusely disposed in the dermis. The mast cells had medium size and their number was similar to the control group. The interstitial edema was moderate in the EM2 1% treated group and mild congestion was observed in the blood vessels, some of them displaying leukocytes margination.

Figure 8I illustrates the skin sample of the group exposed to EM2 and ZnCl_2_ 1%—the number of mast cells were similar to the group exposed to EM2 and fewer than in the Control group. In the reticular dermis, the mast cells were disposed as in the control group. The neutrophils count was similar to the group treated with EM2 and hyalinization was present in the dermis like in the group treated ZnCl_2_ 1%. Furthermore, the epidermal modifications were comparable to those present in the mice treated with ZnCl_2_ 1%. Blood vessels had mild congestion and moderate interstitial edema, similar to the group treated with EM2.

Application of EM2 and ZnCl_2_ 5% lead to an enlargement of the epidermis due to acanthosis with 6 to 7 layers of keratinocytes covered by 5–6 filaments of keratin (Figure 8J). A reduced number of neutrophils was noted. The mast cells were similar in size and number to those in the control group but were distributed in the whole dermis. The interstitial edema and congestion were alike to those from the EM2 group. Leukocytes margination was noted in few blood vessels. Hyalinization was observed in the reticular and superficial dermis and was comparable to the one in the ZnCl_2_ 5% treated group.

## 3. Discussion

CAM assay is an in ovo model used for several biological investigations such as embryologic, immunological, tissue repair, or drug toxicity [30,31,32], and since the discovery of tumor angiogenesis [33] it is frequently used in cancer research [34]. The advantages of this in ovo technique in terms of time, costs, simplicity, and reproducibility recommend it as a good prescreening assay prior to assessment drug effects in murine models. Currently, it has been increasingly used to study potential chemopreventive effects of plant extracts or natural compounds [35].

The triterpenoid maslinic acid is a more recently investigated compound, which has several promising properties such as anti-inflammatory, antioxidant, cardio-protective, antimicrobial, and antitumor [10]. The antiangiogenic effect of maslinic acid was described by Thakor et al. [16] as reducing in vitro the capillary tube formation and the expression of vascular endothelial growth factor (VEGF). Several studies identified a number of molecular targets involved in the angiogenesis process that were influenced by maslinic acid [36,37]. Maslinic acid reduced cell invasion, intercellular adhesion and migration by decreasing the production of vascular endothelial growth factor (VEGF), epidermal growth factor (EGF), matrix metalloproteinase (MMP)-9, MMP-2, intercel-lular adhesion molecule (ICAM), and E-cadherin in human prostate cancer cells [36]. Other groups showed that maslinic acid could prevent inflammation by the enhancement of several cytokines interleukin (IL)-8, IL−1α, IL−1β, and interferon (IFN)-γ, though stimulating macrophage M1 response involved in anticancer defense activities [38].

To our knowledge, no evaluation of EM2 has been previously performed using the CAM assay. An in ovo CAM protocol was performed herein to evaluate EM2, investigating its degree of toxicity, as well as the implications on the normal angiogenesis.

EM2 did not show any signs of toxicity during the five-day administration period. The normal process of angiogenesis was not altered by the incubation with EM2. Moreover, the specimens treated with EM2 did not present any signs of hemorrhage, which was observed in the case of incubation with DMSO, suggesting possible anti-inflammatory mechanisms of protection. The evaluation of EM2 on the chorioallantoic membrane assay indicated good tolerability and lack of irritation with no impairment of the normal angiogenic process during the stages of development it was tested. Zinc and its salts are known to possess anti-inflammatory properties [39]. After the application on the angiogenic chorioallantoic membrane, the combination between EM2 and ZnCl_2_, especially that with 5% ZnCl_2,_ induced normal development of the CAM vessels, with signs of reparative potential and no proinflammatory or proangiogenic effects. ZnCl_2_ alone reduced the number and branching of new forming vessels, as indicated by Güran et al. through the inhibition of fibroblast growth factor 2 (FGF2) [40], but there were signs of vessel fragility. Thus, the association of the two agents could be beneficial for rapid wound healing exceptionally with less scar formation, without angiogenic stimulation [41].

We further investigated the effects of different EM2 formulations for topical application or in association with ZnCl_2_ using a TPA-induced mouse ear inflammation model. Our data indicated that EM2 exhibited mild inhibitory effects on TPA-induced inflammation by decreasing edema and inflammatory infiltrate in mice ears.

Maslinic acid was previously reported to display in vitro and also in vivo anti-inflammatory effects [42,43]. Huang et al. investigated the anti-inflammatory potential of maslinic acid on rat astrocyte cultures stimulated with lipopolysaccharide. The authors stated that the compound inhibited the production of nitric oxide and tumor necrosis factor alpha (TNF-α), events directly correlated with a significant anti-inflammatory effect [42].

Moreover, in an in vivo model of ear edema, the triterpenoid maslinic acid obtained from *Eriobotrya japonica* (Thunb.) Lindl. Reduced TPA-induced inflammation (ID_50_ = 0.13 mg/ear) [43]. It was reported that the compound elicited a stronger anti-inflammatory effect than indomethacin (ID_50_ = 0.3 mg/ear). In another experiment performed by Banno et al. [44], it was indicated that various triterpene acids, from the ursane type (ursolic acid, corosolic acid, pomolic acid, tormentic acid, and hyptadienic acid) and from the oleanane type (oleanolic acid, augustic acid, and 3-epimaslinic acid) display anti-inflammatory activity. Among the compounds, 3-epimaslinic acid elicited a significant reduction of TPA-induced inflammation (ID_50_ = 0.1 mg/ear) compared to indomethacin (ID_50_ = 0.3 mg/ear) [44].

Previous studies about EM2 indicated that the compound had a significant antitumor activity and selectivity in vitro, having positive effects against human melanoma cells (518A2 [12] and A375, murine melanoma cells), B164A5 [45], human thyroid cancer cells (8505C, human ovarian cancer cells), A2780, human breast cancer cells (MCF-7, human lung carcinoma cells), A549, human colorectal adenocarcinoma cells (DLD1, human liposarcoma), Lipo, and human colorectal adenocarcinoma cells (HT29) [12]. EM2 has been previously evaluated by our group in vivo on a rat model of turpentine oil-induced acute inflammation [46]. The compound was orally given before and after inflammation induction at a dose of 50 mg/kg body weight/day during a 10 days period. Both prophylactic and curative treatments provoked a mild reduction of oxidative stress parameters compared to the untreated group. Moreover, in the case of prophylactic administration, a decrease of malondialdehyde comparable to diclofenac was observed [46].

In the present study, we demonstrated that EM2 has mild anti-inflammatory effects, but the effect was stronger when EM2 was associated with ZnCl_2_, especially in high concentrations for the latter, namely 5%. Histological assessment of mice ears showed a decrease in dermal thickness when EM2 and ZnCl_2_ were applied separately or in combination. However, hyalinization of the dermis appeared only when EM2 was associated with ZnCl_2_, indicating that this formulation is not just a potent anti-inflammatory agent but also an important promoter of wound healing.

It is estimated that approximately 9% of the zinc from the human body can be associated with the skin, more specific with the epidermis [27]. Emri et al. also stated that systemic or topic zinc up-take can ameliorate various inflammatory skin conditions [27]. Zinc is used with a high prevalence in daily dermatological products, thus knowing the effects of this microelement at skin level is very important [47]. Both elemental zinc and in the form of salts were reported to induce beneficial effects in patients with skin pathologies. Furthermore, zinc deficiency was associated with acrodermatitis enteropathica among other syndromes [47]. In a clinical trial, a 5% zinc sulphate solution was topical applied to patients with acne vulgaris [48] and compared to another group where a 2% tea lotion was used. After two months of twice a day application, the inflammatory lesions were evaluated. The authors concluded that the tea lotion induced a significant reduction of the inflammatory lesions while the 5% zinc sulphate solution decreased inflammation but at a lower level compared to the tea lotion [48]. In another clinical study [49], zinc sulphate was orally given to patients with rosaceae, a skin condition that affects people worldwide. Zinc sulphate was orally given 100mg/3x/day for three months. Improvement of skin aspect was visible after one month of treatment and, after three months, the erythema was reduced and there were no more papules and pustules. Zinc in its various forms has proven to have beneficial effects in dermatological pathologies. In a recent study, a rutin-zinc(II) complex was evaluated in terms of biological activity [50]. It was stipulated that the flavonoid-metal complex elicited a higher antioxidant effect compared to rutin alone and did not display a cytotoxic activity against normal cells but showed cytotoxic effects when tested on tumor cells (human acute myeloid leukemia cells, multiple myeloma cells, and melanoma cells). Furthermore, after in vivo assessment of the complex toxicity, no significant changes were recorded in treated mice compared to the untreated controls. These results suggest that a combination of an active compound and zinc can lead to a synergistic effect, and might also shorten the duration of a treatment, aspects that directly benefit patients.

Suarez Carmona et al. indicated that cancer can be considered a wound that never heals, suggesting a connection between inflammation and cancer, so finding an active treatment for inflammation could also benefit cancer patients [51].

## 4. Materials and Methods

### 4.1. Chemicals and Reagents

**Figure 9:**
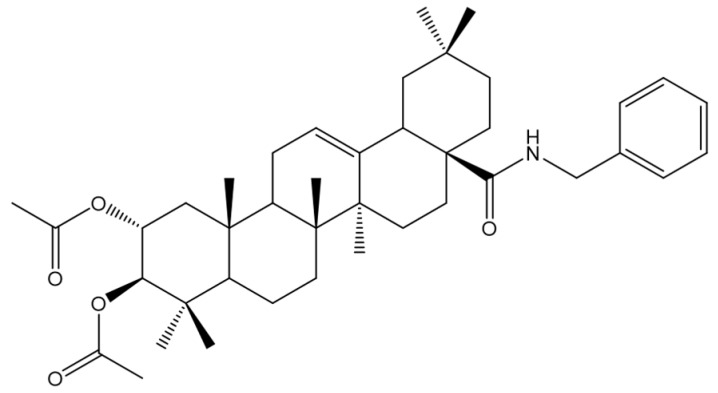
Chemical structure of EM2.

The chemical structures of EM2 is presented in Figure 9. EM2 was synthesized in the Department of Organic Chemistry at Martin-Luther University Halle-Wittenberg [12] and was generously provided by Prof. René Csuk.

TPA was acquired from Sigma-Aldrich (Steinheim, Germany). For the experimental protocol the substance was dissolved in acetone at a concentration of 15 nM TPA. Acetone (analytical purity 99.92%) was purchased from Chimreactiv SRL (Bucharest, Romania). Hydroxypropylmethylcellulose (HPMC), propylene glycol, indomethacin, and the preservative solution were acquired from Chimreactiv SRL (Bucharest, Romania). ZnCl_2_ was acquired from Sigma-Aldrich (Germany).

### 4.2. Transmission Electron Microscopy (TEM) Analysis

In order to determine details about the morphology, holography and crystallization of EM2 microparticles, transmission electron microscopy was employed. For this technique a FEI Tecnai 12 Biotwin microscope was used.

### 4.3. Scanning Electron Microscope (SEM) and Energy Dispersive x-ray (EDAX) Analysis

EM2 and EM2 + ZnCl_2_ 5% microparticles were used for investigation of ultrastructural details regarding the morphology of microparticles surface and its composition, using scanning electron microscopy with energy dispersive X-ray analysis detection (SEM-EDAX). In order to obtain an efficient EM2 + ZnCl_2_ 5% mixture, in a mass ratio of 1:5, the solid compounds were dissolved in a hydro-alcoholic solution, which was sonnicated for 5 min. The solution obtained was then centrifuged at 21,000× *g* at 4 °C for 20 min. The pellet was dehydrated in an oven at 70 °C for 1 h and then the mixture was analyzed by SEM-EDAX. The equipment used for this analysis was an EDAX detector (ZAF Quantification – Standardless, Element Normalized) with an FEI Quanta 250 microscope. The microscope parameters were HV mode, 30kV, ETD (Everhart-Thornley detector for secondary electrons), and two magnification orders; one for a general overview image/measurements and another for higher surface topography sides analysis. The composition of microparticles was expressed in weight percent (Wt %) and atomic percent (At %).

### 4.4. Thermogravimetry (TG) and Differential Scanning Calorimetry (DSC) Analysis

Thermogravimetric and differential scanning calorimetry analysis were employed to elucidate the thermal behavior of the microparticles. The analysis was conducted in air atmosphere at a flow rate of 20 mL·min^−1^ in the range 25–1000 °C with a heating rate of 10 K·min^−1^, using alumina crucibles and a Netzsch STA 449C instrument.

### 4.5. Chorioallantoic Membrane Assay (CAM)

#### Normal Angiogenesis on Chorioallantoic Membrane Assay

EM2 was dissolved in dimethyl sulfoxide (DMSO, Sigma Aldrich) in order to obtain a 10 mM stock solution and further diluted to a concentration of 100 µM prior to use. The solvent DMSO, at a concentration of 1% was used as control. ZnCl_2_ was dissolved in distilled water to a final concentration of 1% (*m*/*m*) or 5% (*m*/*m*) in raport to EM2. Both ZnCl_2_ alone and the mixtures of ZnCl_2_ + EM2 were tested.

CAM assay implies the use of fertilized hen (*Gallus gallus domesticus*) eggs. Using a slightly modified protocol developed by Ribatti et al. [52], the eggs were disinfected and incubated at 37 °C and controlled humidity [53]. On the third embryonic day of development (EDD), 3 to 4 mL of albumen are removed and a window was cut on the upper side of the eggs on the following day (EDD 4). Eggs were returned to incubate until the beginning of the experiment.

EM2 was tested on the normal developing chorioallantoic membrane in order to evaluate the tolerability and the influence on the physiologically enhanced angiogenesis process between EDD 7 and EDD 11 [54]. Samples were applied in volumes of 5 µL on EDD 8 (day 0 of the experiment) directly onto the chorioallantoic membrane inside a plastic ring placed on top of the CAM. EM2 was tested in concentration of 100 µM in diluted DMSO, next to the solvent control. ZnCl_2_ 1% and 5% were tested in the mass ratio of the compound (*m*/*m*). Samples were applied daily for five days until EDD 12.

Evaluation was performed daily in ovo, by means of a stereomicroscope (Zeiss Axio V16 Stereomicroscope, Göttingen, Germany). Images were recorded using the Zeiss Axio Cam digital camera and processed by Zeiss ZEN software (AxioVision SE64. Rel. 4.9.1 Software, Göttingen, Germany).

### 4.6. Preparation of EM2 and ZnCl_2_ Formulations

EM2 was incorporated in a hydrogel formulation in order to overcome the poor solubility of the compound and to promote a better drug delivery system.

The hydrogels were prepared by mixing EM2 and/or ZnCl_2_, hydroxypropyl methylcellulose (HPMC) and propylene glycol, following by the addition of a preservative solution at the end (Table 3). Blank hydrogel (used as ointment base) without the samples (EM2 or ZnCl_2_) was prepared according to a similar process.

### 4.7. In Vivo TPA-Induced Ear Inflammation Protocol

The experiments were performed on SKH1 female mice (weighing 25–29 g) were purchased from Charles River, Budapest, Hungary.

This study is consistent with the Directive 2010/63/EU regarding the protection of animals used for scientific aims. The experimental design was approved by the Ethics Committee of the *"Victor Babes"* University of Medicine and Pharmacy of Timisoara, Romania. Mice were kept under standard conditions (constant temperature of 22.5 ± 2 °C and humidity of 55 + 5%, 12h light/dark cycle) and were fed ad libitum. At the end of the experiment the animals were anesthetized and sacrificed.

The experimental model of TPA-induced ear inflammation was performed according to the following protocol: mice were anesthetised with Sevorane and the TPA solution was topically applied (20 µl/ear). Thirty minutes after applying the TPA solution, mice were randomly assigned to the subsequent groups (Table 4):

An electronic calliper was used to measure mice ears and the results obtained were reported in millimeters. Measures were taken before TPA application: 0 h and at 4 h after TPA application. At the end of the experiment, mice were anesthetized and sacrificed. Ears were collected and maintained in formalin for histopathological evaluation.

### 4.8. Histopathological Assessment of Mice Ears

In order to perform the histopathological analysis, the ears were fixed in 10% buffered formalin and embedded in paraffin after processing. Four micrometer-thick sections from the ears were cut using a Leica Rotary Microtome (Leica Biosystems Nussloch GmbH, Nussloch, Germany). Afterwards, the sections were attached on microscopic slides and were stained with haematoxylin and eosin (H-E staining) for diagnostic purpose. A Leica Light Microscope DM750 was used for the microscopic examination and images were taken with a Leica DMShare System.

## 5. Conclusions

The results of the present study indicate that EM2 has a good tolerability and did not impair the normal angiogenesis process. Moreover, in the TPA-murine ear inflammation model, application of EM2 alone induced a mild reduction of the inflammatory process, an effect that was potentiated by the association EM2 with a high dose of ZnCl_2_. In particular, their concomitant application favored the hyalinization of the dermis, strongly suggesting the role of their combination in wound healing.

## Figures and Tables

**Table 1:** Elemental composition of EM2 [29].

Element	Wt %	At %	K-Ratio	Z	A	F
**C K**	86.95	90.59	0.6886	1.0043	0.7885	1.0001
**N K**	1.17	1.05	0.0009	0.9956	0.0778	1.0001
**O K**	10.58	8.28	0.0130	0.9876	0.1241	1.0000
**Au M**	1.30	0.08	0.0147	0.6616	1.7150	1.0000
**Total**	**100.00**	**100.00**				

**Table 2:** Elemental composition of EM2 + ZnCl_2_.

Element	Wt %	At %	K-Ratio	Z	A	F
**C K**	88.94	92.87	0.5971	1.0046	0.6682	1.0001
**O K**	8.29	6.50	0.0092	0.9905	0.1123	1.0000
**Zn L**	2.15	0.41	0.0109	0.8490	0.5946	1.0000
**Cl K**	0.61	0.22	0.0060	0.9108	1.0682	1.0000
**Total**	**100.00**	**100.00**				

**Table 3:** EM2 and ZnCl_2_ formulations [29]. HPMC: hydroxypropyl methylcellulose.

Formulations		EM2 (mg)	ZnCl_2_ (mg)	HPMC (mg)	Propylene Glycol (mg)	Preservative Solution (mL)
1.	EM2 1%	1	−	2	10	87
2.	ZnCl_2_ 1%	−	1	2	10	87
3.	ZnCl_2_ 5%	−	5	2	10	83
4.	EM2 1% + ZnCl_2_ 1%	1	1	2	10	86
5.	EM2 1% + ZnCl_2_ 5%	1	5	2	10	82
6.	Blank hydrogel	−	−	2	10	88

**Table 4:** In vivo experimental design for the local acute inflammation model.

Group No.	Group Name	Group Description
1	**Control**	With no intervention
2	**Acetone**	Acetone solution application (20 µl/ear)
3	**TPA**	TPA solution application (20 µl/ear)
4	**TPA + indomethacin**	A 4% indomethacin cream was topically applied 30 min after applying the TPA solution
5	**Blank hydrogel**	Blank hydrogel was topically applied 30 min after TPA solution application
6	**EM2 1%**	EM2 1% hydrogel was topically applied 30 min after TPA solution application
7	**ZnCl_2_ 1%**	ZnCl_2_ 1% hydrogel was topically applied 30 min after TPA solution application
8	**ZnCl_2_ 5%**	ZnCl_2_ 5% hydrogel was topically applied 30 min after TPA solution application
9	**EM2 1% + ZnCl_2_ 1%**	EM2 + ZnCl_2_ 1% hydrogel was topically applied 30 min after TPA solution application
10	**EM2 1% + ZnCl_2_ 5%**	EM2 + ZnCl_2_ 5% hydrogel was topically applied 30 min after TPA solution application

## Abbreviations

CAM	Chorioallantoic membrane
DMSO	Dimethyl sulfoxide
EDAX	Energy dispersive X-ray analysis
EDD	Embryonic day of development
EM2	Benzyl (2α, 3β) 2,3-diacetoxy-olean^−1^2-en-28-amide
H-E	Hematoxilin-eosine
HPMC	Hydroxypropylmethylcellulose
SEM	Scanning electron microscope
TEM	Transmission electron microscopy
TG-DSC	Thermogravimetry-differential scanning calorimetry
TNF-α	Tumor necrosis factor alpha
TPA	12-o-tetradecanoylphorbol^−1^3-acetate
VEGF	Vascular endothelial growth factor
ZnCl_2_	Zinc chloride
